# Centrality of G6PD in COVID-19: The Biochemical Rationale and Clinical Implications

**DOI:** 10.3389/fmed.2020.584112

**Published:** 2020-10-22

**Authors:** Yuliya Buinitskaya, Roman Gurinovich, Clifford G. Wlodaver, Siarhei Kastsiuchenka

**Affiliations:** ^1^Sci.AI, Tallinn, Estonia; ^2^Oklahoma University Health Sciences Center, Oklahoma City, OK, United States; ^3^Anesthesiology Institute, Cleveland Clinic Abu Dhabi, Abu Dhabi, United Arab Emirates

**Keywords:** COVID-19, glucose-6-phosphate dehydrogenase (G6PD), reactive oxygen species, nitric oxide - NO, glutathione, aldosterone (Ald), Metabolic syndrome

## Abstract

**Introduction:** COVID-19 is a novel and devastating disease. Its manifestations vary from asymptomatic to lethal. Moreover, mortality rates differ based on underlying health conditions and ethnicity. We investigated the biochemical rationale behind these observations using machine reasoning by the sci.AI system (https://sci.ai/). Facts were extracted and linked from publications available in nlm.nih.gov and Europe PMC to form the dataset which was validated by medical experts.

**Results:** Based on the analysis of experimental and clinical data, we synthesized detailed biochemical pathways of COVID-19 pathogenesis which were used to explain epidemiological and clinical observations. Clinical manifestations and biomarkers are highlighted to monitor the course of COVID-19 and navigate treatment. As depicted in the Graphical Abstract, SARS-CoV-2 triggers a pro-oxidant (PO) response leading to the production of reactive oxygen species (ROS) as a normal innate defense. However, SARS-CoV-2's unique interference with the antioxidant (AO) system, through suppression of nitric oxide (NO) production in the renin- angiotensin-aldosterone system (RAAS), leads to an excessive inflammatory PO response. The excessive PO response becomes critical in cohorts with a compromised AO system such as patients with glucose-6-phosphate dehydrogenase deficiency (G6PDd) where NO and glutathione (GSH) mechanisms are impaired. G6PDd develops in patients with metabolic syndrome. It is mediated by aldosterone (Ald) which also increases specifically in COVID-19.

**Conclusion:** G6PD is essential for an adequate immune response. Both G6PDd and SARS-CoV-2 compromise the AO system through the same pathways rendering G6PDd the Achilles' heel for COVID-19. Thus, the evolutionary antimalarial advantage of the G6PDd cohort can be a disadvantage against SARS-CoV-2.

**Graphical Abstract:**
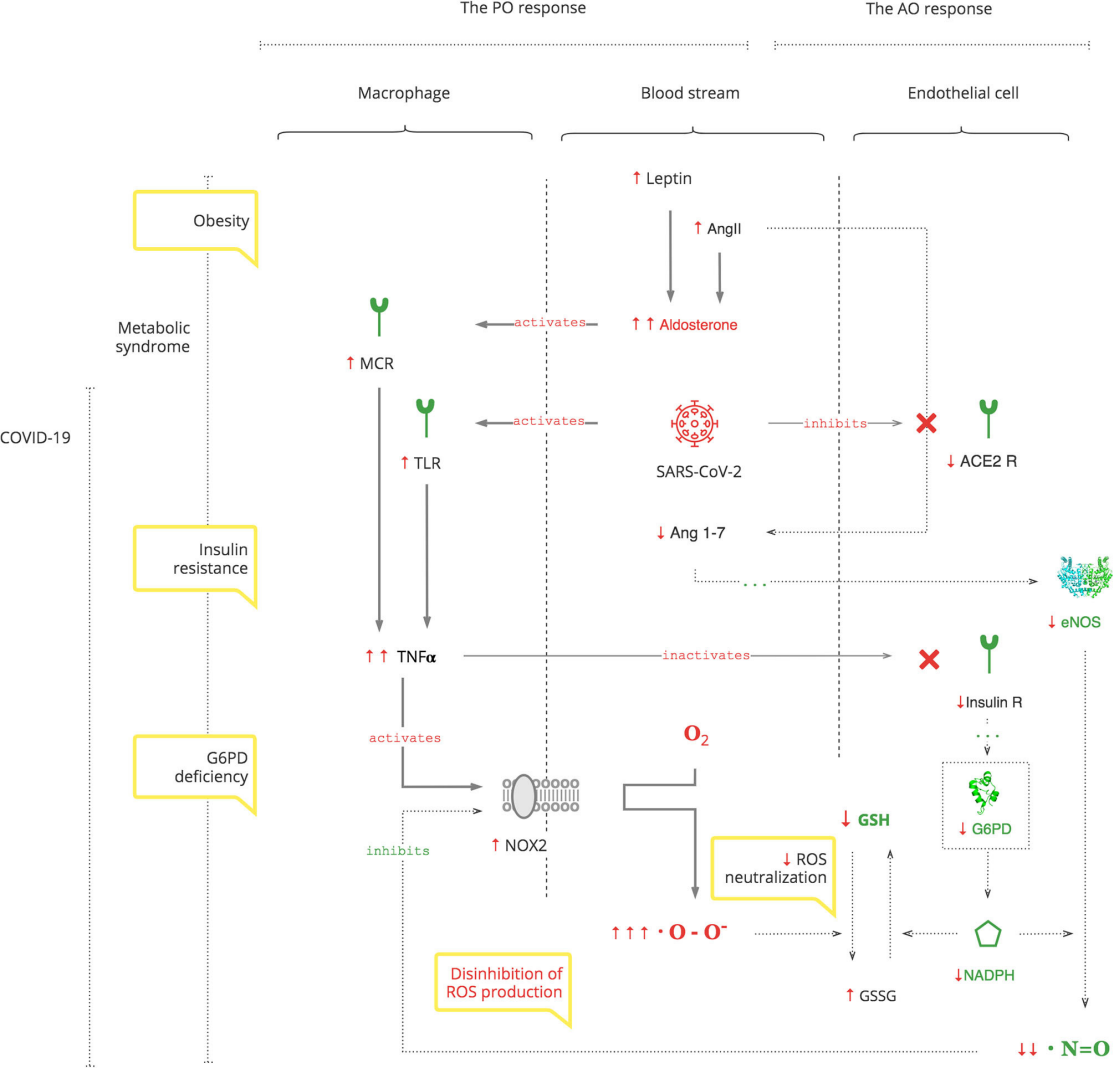


## Introduction

Based on our previous discussion about the basic mechanisms of coronavirus disease 2019 (COVID-19) pathogenesis (1), in this paper, we will focus on particular parts of it to explain the present clinical and epidemiological observations.

The human host defends itself against infection through its immune response with two cooperative phases.

1. The first phase occurs early, is innate and nonspecific, and has two components.

- A pro-inflammatory pro-oxidant (PO) system mediates inflammation. It attacks pathogens with free radicals of the reactive oxygen species (ROS). However, when there is increased production and/or decreased neutralization, a heightened level of ROS occurs, and excessive levels cause collateral damage to normal cells and is referred to as “oxidative stress,” “cytokine storm,” and “systemic inflammatory response syndrome (SIRS).”
- An anti-inflammatory antioxidant (AO) system balances the PO response (2).

2. The second phase occurs with a delay and is adaptive and specific. It is mediated through antibody expression.

These two arms of immune response usually eradicate the pathogen (3). However, in COVID-19, both phases are delayed due to suppression of the host's gene expression by the severe acute respiratory syndrome coronavirus 2 (SARS-CoV-2)'s nsp1 protein (4).

Wu et al. (5) demonstrated, *in vitro*, that glucose-6-phosphate dehydrogenase deficiency (G6PDd) cell lines are vulnerable to coronavirus infection. There are two types of G6PDd: congenital and acquired. Congenital G6PDd is the most prevalent enzyme deficiency in the world, affecting 4.9% of the global population. It evolved against malaria and predominates in specific ethnic cohorts such as the Mediterranean, Asian, and African (6). Interestingly, these cohorts have been particularly affected by the COVID-19 pandemic (7–10). Acquired G6PDd develops in patients with underlying health conditions, especially the metabolic syndrome (11). The metabolic syndrome is prevalent and it spreads acquired G6PDd worldwide.

This paper presents a detailed description of how SARS-CoV-2 affects the innate PO and AO responses and how G6PDd potentiates COVID-19. In addition, we highlight accompanying clinical manifestations and biomarkers that are useful to monitor the clinical course and navigate treatment.

## Methods

We used the sci.AI machine reasoning system (https://sci.ai/) to operate on publicly available datasets from nlm.nih.gov and Europe PMC. The process consisted of two stages: Representation and Reasoning.

Representation algorithms translate unstructured individual papers, documents, and files from heterogeneous sources into embeddings and graphs of entities relations. It goes beyond classic Named Entity Recognition (NER) and arbitrarily recognizes individual and composite biological entities and how they relate to each other. For example, in this sentence: “Obese patients with MetSyn had a significantly lower nitric oxide production rate (0.21 ± 0.13 μmol/h per kg; *P* = 0.009) than healthy normal-weight individuals (0.63 ± 0.30 μmol/h per kg), whereas nitric oxide (NO) production rate was intermediate in obese patients without MetSyn (0.49 ± 0.22 μmol/h per kg; *P* = 0.33)” (12); the machine recognizes the conditions “obesity” and “metabolic syndrome” and recognizes substance “nitric oxide” and links it to CHEBI:16480. Ultimately, “lower nitric oxide production rate” in the context of “metabolic syndrome” is recognized as a biomarker.

The second, Reasoning stage, synthesizes knowledge based on a subset of findings that appear to be relevant to COVID-19. The discovery process was triggered by textual queries “SARS” and “ARDS.” Traversing through the interlinked representations computed at the first stage produced multiple subgraphs. We progressively refined the generated knowledge and, in the last step, linked these excerpts to synthesize biochemical pathways to help explain the pathophysiology of COVID-19. We translated complex pathways into clinically relevant applications, conforming to our clinical observations.

Pathways were constructed iteratively; it is not a result of one time inference. Generally speaking, typical machine learning algorithms approximate previous data distributions. In contrast, our reasoning algorithm is based on graph traversing and utilizes biochemical properties in context. It allows to avoid bias caused by frequently mentioned terms, for example, angiotensin-converting enzyme 2 (ACE2). Subgraphs were interactively validated by a domain expert at every iteration. For instance, the term “SARS” mentioned together with “TLR” and “ACE2” led to the creation of two axes as described in our previous work (1):

– TLR/TNFα/NADPH oxidase (NOX2)/ROS, which is positively regulated, and

– ACE2/NOS3/NO, which is negatively regulated by SARS.

Both axes turn out to be composed mainly of canonical pathways: renin–angiotensin system, glutathione (GSH) metabolism, pentose phosphate pathway, aldosterone (Ald) synthesis and secretion, and NO production. When we placed all these pathways on the same canvas, reduced nicotinamide adenine dinucleotide phosphate (NADPH) appeared to be the cofactor of both axes and, in turn, is produced by glucose-6-phosphate dehydrogenase (G6PD). This biochemical rationale, together with the worldwide prevalence of congenital and acquired G6PDd, is consistent with COVID-19 outcomes at individual and epidemiological levels.

A limitation of our research is that it focuses on the centrality of G6PD. Yet, we acknowledge that there is certainly other biochemistry relevant to COVID-19 that remains open for investigation.

## Results and Discussion

Based on machine reasoning of data from 30M papers, we demonstrate the results.

### Severe Acute Respiratory Syndrome Coronavirus 2 Affects the Innate Immune Response

#### The PO System

**Figure 1:**
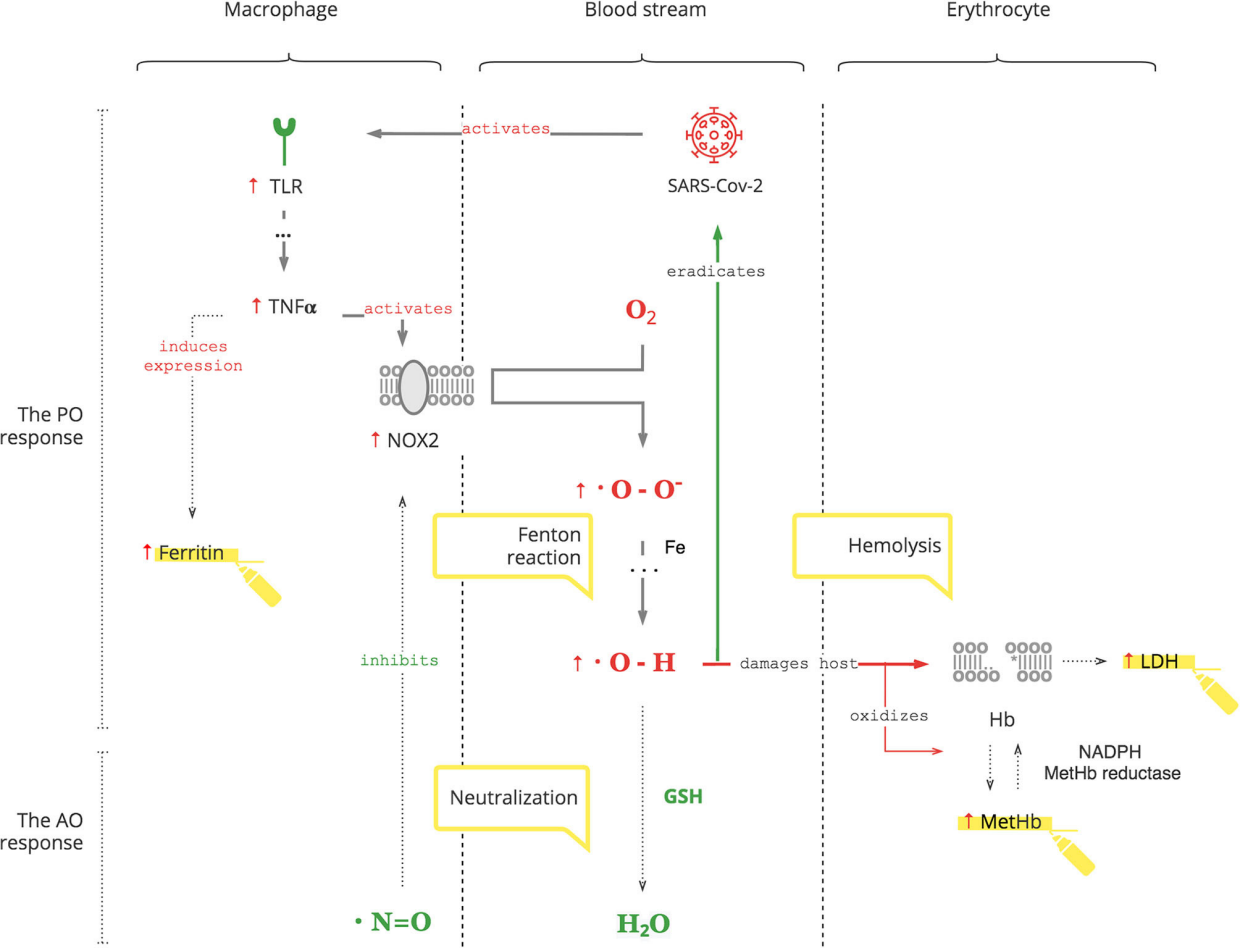
SARS-CoV-2 triggers the PO response resulting in ROS.

*The PO system* is triggered by SARS-CoV-2, as for any pathogen, through toll-like receptors (TLRs) on macrophages, the first-line cell of innate defense (13). As depicted in Figure 1, this results in tumor necrosis factor-alpha (TNFα)-induced inflammation, which has two clinically relevant molecular effects: inactivation of insulin receptor signaling on endothelial cells (see Graphical Abstract) and activation of NOX2 on macrophages (14, 15). This response is acute and transient. Activated NOX2 produces ROS, particularly superoxide anion (O2^*^-) from oxygen (O2). Then a hydroxyl radical (OH^*^) is produced through the Fenton reaction (16). It destroys microorganisms (17). At the same time, it stresses the host's cells, especially platelets, lymphocytes, erythrocytes, and muscle cells (18–20). Muscle cell damage manifests with rhabdomyolysis (21). Damage of erythrocyte membranes results in latent hemolysis leaking lactate dehydrogenase (LDH), and hemoglobin (Hb) is oxidized to methemoglobin (MetHb) (22–24).

#### Clinical Pearls

- Hyperglycemia occurs during COVID-19. It is transient and reversible if there is no antecedent insulin resistance. Otherwise, underlying insulin resistance is aggravated by the stress of infection (25).
- Ferritin production is induced by TNFα and can be used to monitor the degree of the PO response (26).
- Thrombocytopenia and lymphopenia reflect the degree of oxidative stress and can be followed as biomarkers (27, 28).
- Since statins cause rhabdomyolysis as a complication, avoid these drugs in COVID-19 patients (29, 30).
- Erythrocytes are decreased due to latent hemolysis, which can be monitored by LDH levels (31).
- Increased MetHb makes SpO_2_ calculation inaccurate. It causes a low SpO_2_ by pulse oximetry in patients with a normal PaO_2_ (32, 33). This can be misleading and can result in an unnecessary administration of O_2_, the substrate of ROS.

Thus, SARS-CoV-2 interacts with the innate PO system, and adequate ROS levels are a first-line antimicrobial defense.

#### The AO System

*The AO system* balances the PO response through two central mechanisms: suppression of ROS production by NO and ROS neutralization by GSH (34, 35).

**Figure 2:**
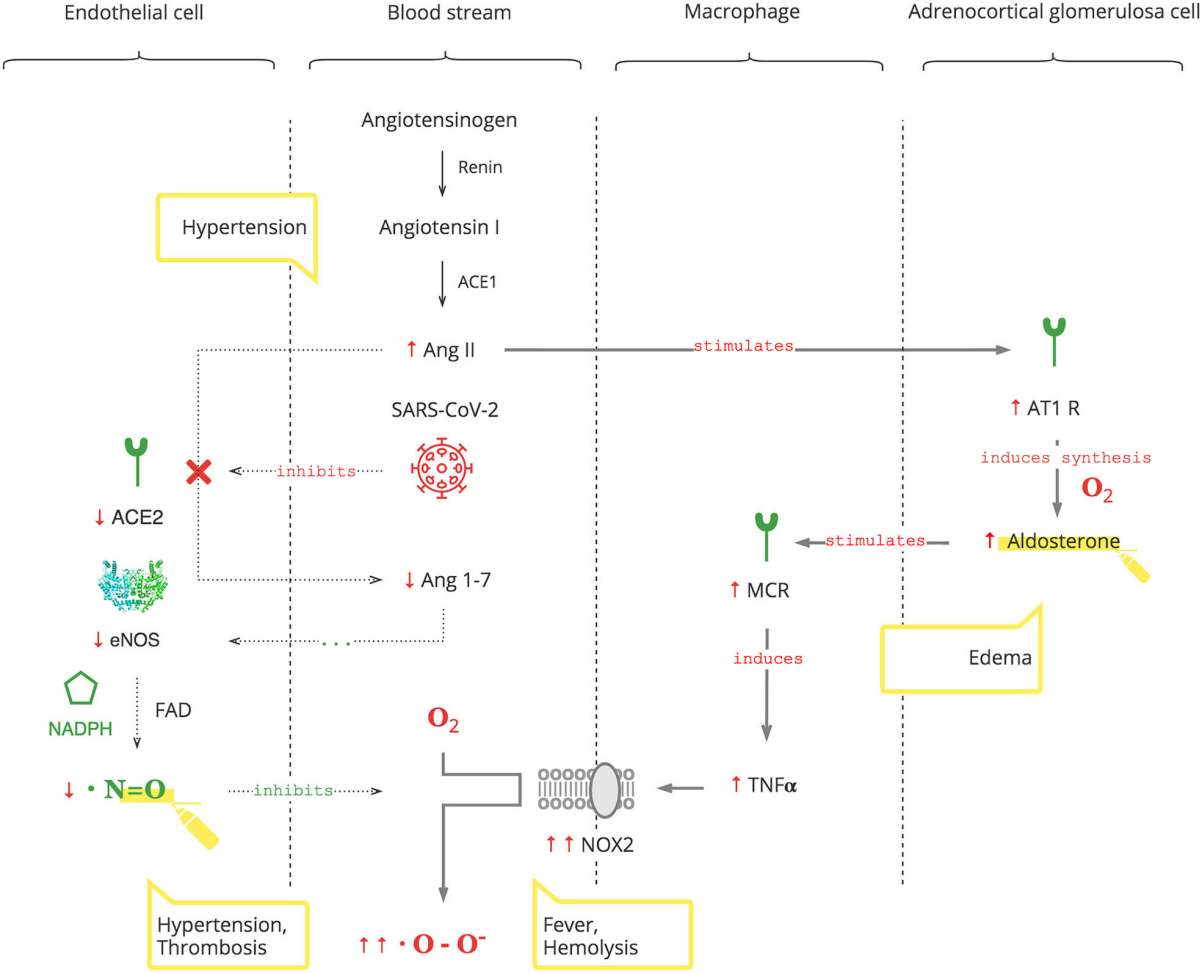
SARS-CoV-2 interferes with the AO response through RAAS resulting in excessive ROS.

As depicted in Figure 2, SARS-CoV-2 binds to the ACE2 receptor in order to enter cells and, in turn, destroys this receptor. The ACE2 receptor is involved in the protective ACE2/endothelial nitric oxide synthase (eNOS)/NO pathway of the renin–angiotensin–aldosterone system (RAAS). It leads to the suppression of eNOS, the most prevalent isoform of NOS, and consequently decreased NO levels (36). In addition to its antioxidant property, NO is also essential for vasodilation, prevention of platelet aggregation, and inhibition of the replication of SARS-CoV (37, 38).

Suppression of ACE2 activity also leads to an inability to convert angiotensin II (Ang II) to angiotensin 1-7 (Ang 1-7). Ang II is a potent vasoconstrictor and also stimulates Ald (39). This results in a transient increase in Ald that induces TNFα through mineralocorticoid receptor (MCR) on macrophages (40). NOX2 hyper-activation by TNFα, which is induced by the virus and Ald, and its disinhibition by virus-induced NO inhibition perpetuate ROS production, making it excessive.

It is also noteworthy that adrenocortical glomerulosa cells are extremely sensitive to dissolved O_2_ blood levels (41). And fever shifts the O_2_-Hb dissociation curve to the right, lowering the affinity of Hb to O_2_, further contributing to ROS production (42).

#### Clinical Pearls

- An increased Ald level is a specific biomarker of the SARS-CoV-2 infection. This is acute, transient, and Ang II-dependent.
- The virus-induced decrease in NO and increase in Ald (NO/RAAS dysbalance) render the immune response to COVID-19 excessive. This manifests with fever and hematological complications, especially progressive hemolysis, and thrombus formation (43).
- Vasoconstriction, mediated by NO/RAAS dysbalance, is a main pathophysiological component of COVID-19-associated acute respiratory distress syndrome (ARDS) and manifests as acute vascular distress syndrome (AVDS) (44).
- Pulmonary edema is potentiated by elevated levels of Ald and aggravates ARDS (45).
- Excessive O_2_ therapy can be deleterious.

Thus, SARS-CoV-2 interferes with the AO system, rendering the PO response excessive. Moreover, SARS-CoV-2-induced increases of Ald aggravate the condition, especially in patients with underlying health conditions.

### The Role of Underlying Health Conditions

Individuals probably contract COVID-19 at similar rates. However, once infected, some persons do worse than others. The inoculum of infection may be an important variable (46) but will not be further discussed here. We will focus on the role of underlying health conditions. And we will relate these to the PO and AO immune responses that we discussed above.

As noted above, COVID-19 induces an excessive PO response. This needs to be balanced by AO mechanisms: NO and GSH. As depicted in Graphical Abstract, these two mechanisms are dependent on the cofactor NADPH (47, 48). It is produced mainly by G6PD in a rate-limiting manner in the pentose phosphate pathway (PPP) of glucose metabolism. In addition to NO and GSH, there are several other systems that require NADPH and compete for it: macrophage NADPH oxidase (NOX2) for antimicrobial defense, NADPH methemoglobin reductase for Hb recovery, and thyroid NADPH oxidase for triiodothyronine (T3) production (49). The inability of G6PD to supply enough NADPH for the excessive immune response, along with these other demands, aggravates G6PDd. Thus, G6PD is essential for both components of innate immune response and, particularly, for the AO system to balance the PO system (50).

NO and GSH are also dependent on flavin adenine dinucleotide (FAD). FAD production is catalyzed by T3, which requires NADPH for its synthesis by thyroid NADPH oxidase (51). Thus, G6PDd ultimately decreases T3, NO, and GSH, thereby compromising the body's defensive mechanisms.

#### Acquired G6PDd

While congenital G6PDd is well known, its acquired deficiency is less appreciated. It accompanies insulin resistance (52) and hypertension (53, 54), grouped together as the metabolic syndrome. In addition, advancing age also lowers it (55). We demonstrate the biochemical rationale of these findings and why these cohorts do worse with COVID-19.

**Figure 3:**
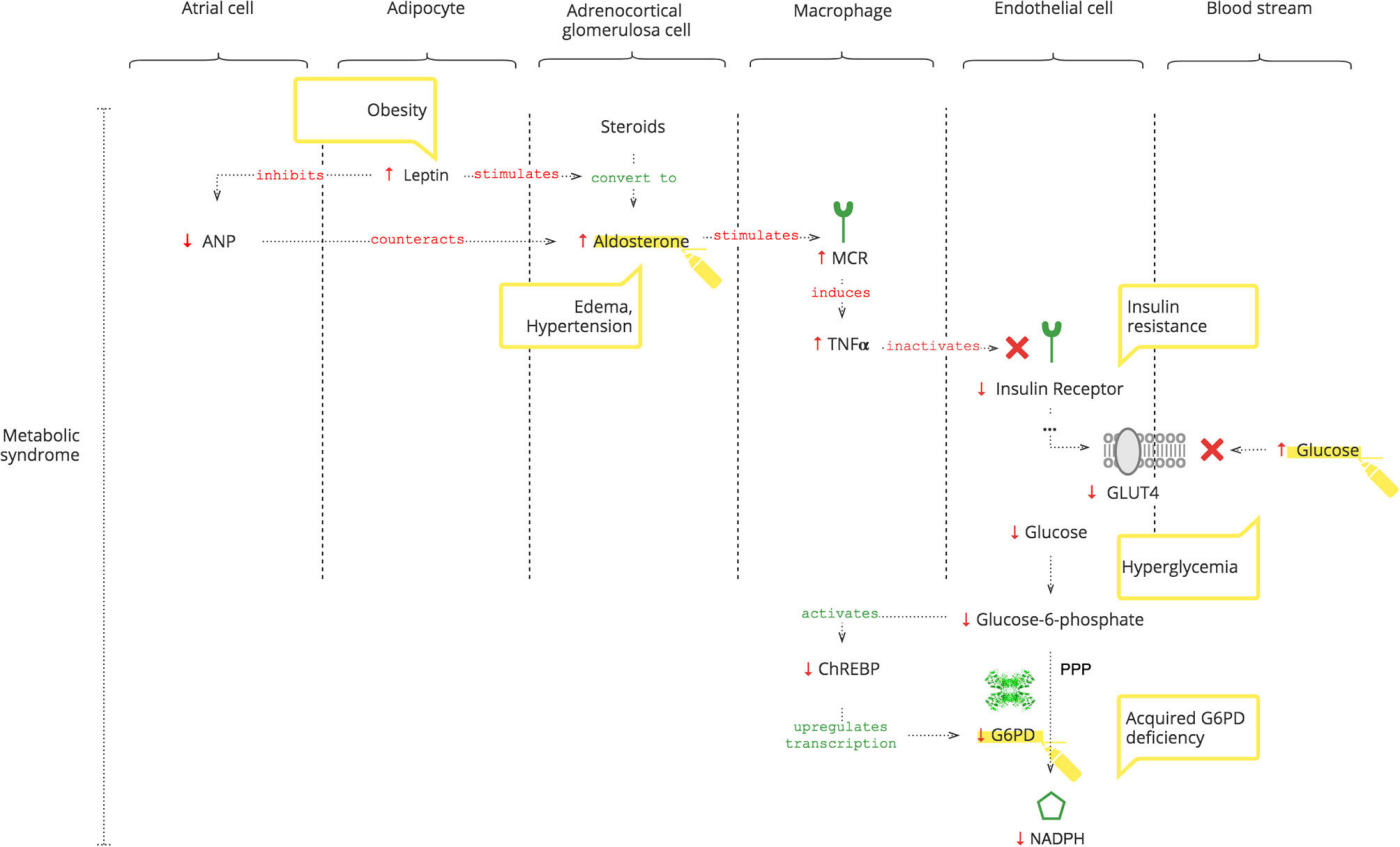
How the metabolic syndrome leads to acquired G6PDd.

As depicted in Figure 3, adipocytes secrete leptin. Obesity-induced hyper-leptinemia is chronic and progressive and directly stimulates Ald (56). Moreover, leptin suppresses atrial natriuretic peptide (ANP), which helps to “escape” Ald activity (57). Increased Ald, as discussed previously, results in increased TNFα (37). In addition to NOX2 activation, chronic TNFα stimulation also causes insulin resistance (14, 58). Under normal conditions, insulin receptor signaling is required for glucose entrance into cells. Glucose is phosphorylated to glucose-6- phosphate, which activates the carbohydrate response element-binding protein (ChREBP) (59). ChREBP regulates the expression of rate-limiting enzymes in glucose metabolism, in particular G6PD (60). Thus, decreased intracellular glucose results in decreased G6PD gene expression and, consequently, lower NADPH (61).

Moreover, Liao et al. (62) showed that there is no significant difference in the expression of TNFα between G6PDd and normal patients.

#### Clinical Pearls

- Even when SARS-CoV-2 can no longer be detected and antibodies have formed, clinical manifestations of inflammation may ensue due to Ald-triggered TNFα.
- Leptin-induced increase of Ald is chronic, progressive, and AngII-independent, and it is not controlled by the RAAS, so angiotensin II receptor blockers (ARBs) and ACE inhibitors can be ineffective as antihypertensives (63).
- Decreased ANP levels in patients with metabolic syndrome render them vulnerable to COVID-19-induced acute increased Ald (64, 65).
- In COVID-19 patients with metabolic syndrome, hypertension, edema, and hyperglycemia accentuate.
- Chronic hyperglycemia can cause insulin resistance and can be a biomarker of developing G6PDd (66, 67).
- Laboratory values of G6PD levels and resulting NADPH activity can differ for several reasons: highly variable glucose level-dependent G6PD gene expression; the unique rate-limiting catalyzation of NADPH production; and the overload of immune mechanisms competing for NADPH, especially in patients with developing G6PDd.
- T3 levels reflect the NADPH activity but also can be involved in thyroid gland disorders.
- Metabolic syndrome-related chronic G6PDd can be aggravated by COVID-19-induced insulin resistance.
- As a consequence, patients with metabolic syndrome have a decreased level of NO and exogenous NO treatment can be considered (12, 68).
- Optimal control of underlying chronic diseases helps defend against COVID-19.

Thus, metabolic syndrome causes G6PDd. And G6PDd, by reducing NO, dysbalances the immune response to COVID-19. In addition, GSH plays a critical role as discussed below.

#### The Role of GSH System

**Figure 4:**
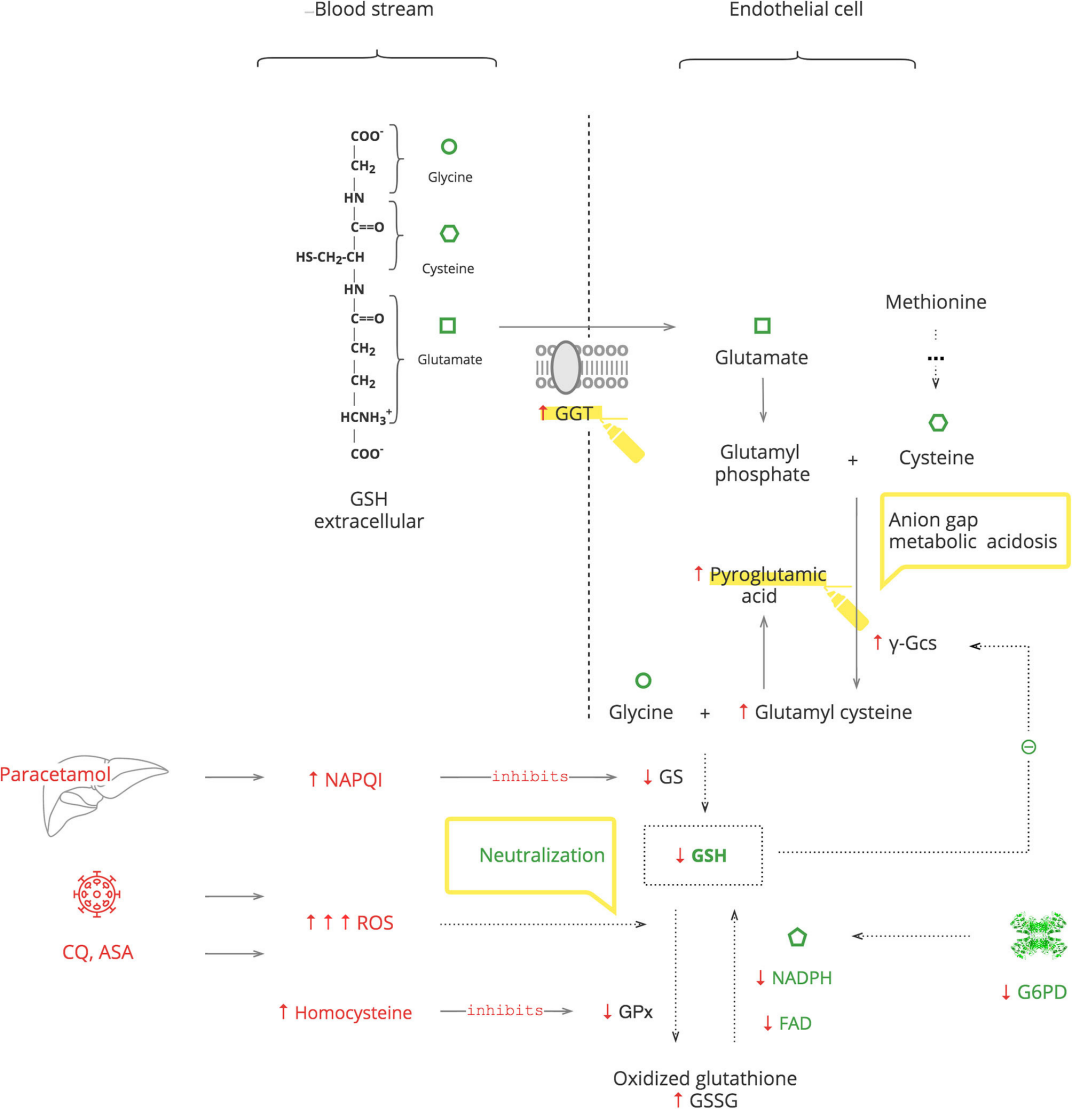
GSH depletion activates mechanisms of GSH repletion.

*GSH* is an essential endogenous antioxidant. As depicted in Figure 4, it is composed of three amino acids: glycine, cysteine, and glutamate. The sulfhydryl (-SH) moiety of cysteine is responsible for the neutralization of toxic substances, both endogenous such as ROS and exogenous such as xenobiotics. During this reaction, GSH is oxidized to its inactive form, GSSG. The recycling requires NADPH and FAD (69).

GSH depletion can be caused by G6PDd, which leads to an inability to recycle it (70, 71). It can also be caused by excessive levels of toxic substances that overload the capacity for its neutralization. Furthermore, the GSH system can be compromised by exogenous substances, e.g., the paracetamol metabolite, N-acetyl-p-benzoquinone imine (NAPQI), which inactivates glutathione synthetase (GS) of GSH production, and by endogenous substances, e.g., homocysteine, which inactivates glutathione peroxidase (GPx) of GSH function (72, 73). The body responds with γ-glutamyl transferase (GGT) upregulation to replete intracellular amino acids from extracellular GSH and also by *de novo* production of cysteine from methionine (74, 75). These amino acids then enter the γ-glutamyl cycle. When there is abundant GSH, it suppresses its own production by blocking γ-glutamyl cysteine synthase (γ-Gcs). Otherwise, GSH depletion results in increased γ-Gcs, leading to accumulation of pyroglutamic acid (76).

#### Clinical Pearls

- Exogenous stresses such as infection, medications, e.g., chloroquine (CQ), aspirin (ASA), and medical procedures, are accompanied by increased ROS production, which exacerbates GSH deficiency (77–80).
- In COVID-19, paracetamol is used as an antipyretic to avoid NSAIDs, and it accentuates GSH deficiency (69).
- Exacerbation of G6PDd manifests with fever and hematologic complications, especially hemolytic anemia. If a patient's Hb decreases after 2–3 days on certain treatments, e.g., CQ or O_2_ therapy, and the LDH level has increased, G6PDd should be considered.
- In critically ill patients, severe G6PDd manifests with transient hypothyroidism also known as the “low T3 syndrome” or the “euthyroid sick syndrome” (81).
- Patients with metabolic syndrome have increased levels of homocysteine. Consider folic acid and/or cyanocobalamin deficiency in these patients to prevent aggravation of GSH depletion (82, 83).
- Severe GSH deficiency clinically manifests with unexplained anion gap metabolic acidosis. This should be considered as pyroglutamic acidosis until proven otherwise. This acidemia, by itself, is not clinically important, but it is a sign of serious metabolic stress (73).
- An increased level of GGT can be used as a biomarker of GSH depletion.

## Conclusion

G6PD activity is essential for the adequate functioning of both the PO and AO components of the innate immune response to counteract COVID-19-induced immune dysregulation. Therefore, in COVID-19 patients, inadequate G6PD activity should be considered and can be monitored with biomarkers. Recognizing these interactions is critical to avoid inappropriate treatment. “*Primum non nocere*.”

## Data Availability Statement

The raw data supporting the conclusions of this article will be made available by the authors, without undue reservation.

## Author Contributions

All authors contributed to the work equally. All authors read and approved the final manuscript.

## Conflict of Interest

YB and RG were employed by sci.AI. The remaining authors declare that the research was conducted in the absence of any commercial or financial relationships that could be construed as a potential conflict of interest.
